# Excessive production and extreme editing of human metapneumovirus defective interfering RNA is associated with type I IFN induction

**DOI:** 10.1099/vir.0.066100-0

**Published:** 2014-08

**Authors:** Bernadette G. van den Hoogen, Sander van Boheemen, Jonneke de Rijck, Stefan van Nieuwkoop, Derek J. Smith, Brigitta Laksono, Alexander Gultyaev, Albert D. M. E. Osterhaus, Ron A. M. Fouchier

**Affiliations:** 1 Department of Viroscience, Erasmus MC, Rotterdam, The Netherlands; 2 Center for Pathogen Evolution, Department of Zoology, University of Cambridge, Cambridge, UK

## Abstract

Type I IFN production is one of the hallmarks of host innate immune responses upon virus infection. Whilst most respiratory viruses carry IFN antagonists, reports on human metapneumovirus (HMPV) have been conflicting. Using deep sequencing, we have demonstrated that HMPV particles accumulate excessive amounts of defective interfering RNA (DIs) rapidly upon *in vitro* passage, and that these are associated with IFN induction. Importantly, the DIs were edited extensively; up to 70 % of the original A and T residues had mutated to G or C, respectively. Such high editing rates of viral RNA have not, to our knowledge, been reported before. Bioinformatics and PCR assays indicated that adenosine deaminase acting on RNA (ADAR) was the most likely editing enzyme. HMPV thus has an unusually high propensity to generate DIs, which are edited at an unprecedented high frequency. The conflicting published data on HMPV IFN induction and antagonism are probably explained by DIs in virus stocks. The interaction of HMPV DIs with the RNA-editing machinery and IFN responses warrants further investigation.

## Introduction

Human metapneumovirus (HMPV), a paramyxovirus in the genus *Metapneumovirus* within the subfamily *Pneumovirinae* (van den Hoogen *et al.*, 2001), is responsible for 5–15 % of hospitalizations of children suffering from acute respiratory tract infections (van den Hoogen *et al.*, 2001; Williams *et al.*, 2004). Phylogenetic analysis has identified two main genetic lineages, with isolates NL/1/00 and NL/1/99 as prototypes of lineages A and B, respectively (van den Hoogen *et al.*, 2004).

As a first line of defence, the innate immune system protects cells against viral infections through production of type I IFNs. For successful infection and replication, viruses use a variety of strategies to evade the host innate immune system (reviewed by Randall & Goodbourn, 2008). Respiratory syncytial virus (RSV), the mammalian pneumovirus most closely related to HMPV, uses two non-structural proteins (NS1 and NS2) as IFN antagonists (Spann *et al.*, 2005). Homologues of NS1 and NS2 are absent in the genome of HMPV (van den Hoogen *et al.*, 2002), indicating that HMPV must have a different strategy to evade the innate immune system. It has been suggested that HMPV is a strong activator of the retinoic acid-inducible gene 1 (RIG-I) and/or mitochondrial antiviral-signalling protein (MAVS) signalling pathways leading to IFN production (Baños-Lara et al., 2013; Bao *et al.*, 2007; Liao *et al.*, 2008). In contrast, the interaction of the attachment protein (G) of HMPV with RIG-I resulted in inhibition of RIG-I activation (Bao *et al.*, 2008). However, using small interfering RNA (siRNA) silencing of G, an antagonistic role of G was not confirmed (Preston *et al.*, 2012). Goutagny *et al.* (2010) reported that only NL/1/00 induced IFN production, in contrast to NL/1/99. Using the same virus strains, we could not reproduce IFN production upon NL/1/00 infection. These discrepancies between different studies led us to hypothesize that defective interfering RNAs (DIs) present in virus stocks may be responsible for the reported activation of the IFN pathway. DIs consist of partially deleted viral genomes that arise spontaneously when virus stocks are generated by passaging at high m.o.i. in mammalian cells, due to errors made by the replicase complex (Huang, 1973). The presence of DIs in virus stocks was reported in the 1960s and their generation by diverse DNA and RNA viruses has been widely documented (Rima *et al.*, 1977; Sekellick & Marcus, 1978; Strahle *et al.*, 2006). DIs with a ‘copyback’ or ‘snapback’ structure are strong IFN inducers, which correlates with their ability to self-anneal and form dsRNA (Sekellick & Marcus, 1978; Strahle *et al.*, 2006). ‘Copyback’ types are generated during generation of the negative-sense strand by the replicase, which leaves its positive-sense template and resumes synthesis at the beginning of the ‘daughter’ genome negative-sense chain that it is still carrying. The complementary ends of the two chains are responsible for the circular ‘panhandle’ structures that these genomes form as naked RNA (Kolakofsky, 1976). ‘Snapback’ DIs are generated in a similar way to the ‘copyback’ DIs; however, the replicase does not only copy back the 3′ end but also, when it crosses to the nascent strand, copies back the complete strand, resulting in a covalently linked positive- and negative-sense strand, forming a duplex (Lazzarini *et al.*, 1981).

Here, we have demonstrated for the first time, to our knowledge, that HMPV accumulates DIs with a snapback structure rapidly upon *in vitro* passage, causing activation of the IFN pathway upon infection. Strikingly, the genomes of each of the DIs displayed extensive A→G or T→C hypermutation. In addition, our data indicated that the RNA-editing enzyme adenosine deaminase acting on RNA (ADAR) was the most likely enzyme responsible for editing of HMPV DIs.

## Results

### IFN production upon HMPV infection

The ability of HMPV to induce IFN production was tested following inoculation of A549 cells with two different virus stocks of recombinant HMPV strain NL/1/00. One stock was generated with a maximum of two passages in Vero-118 cells at low m.o.i. of 0.01 (hereafter named P2_low_), and a second stock was generated by five passages in Vero-118 cells at a high m.o.i. of 3 (hereafter named P5_high_).

**Fig. 1.**
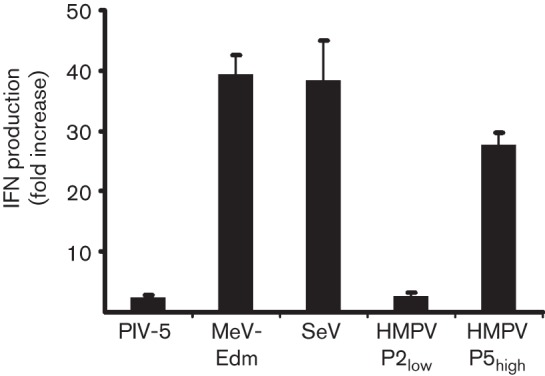
Induction of type I IFN in paramyxovirus-infected A549 cells. Production of IFN in A549 cells induced by PIV-5, MeV-Edm, SeV and HMPV P2_low_ and P5_high_ at 24 h post-inoculation. IFN production was measured using an ISRE-firefly luciferase bioassay. Values indicate fold induction compared with mock-infected cells. The experiment was conducted in duplicate, and a representative of three independent experiments is depicted.

A549 cells were inoculated with these stocks as well as with sendai virus (SeV), measles virus Edmonston strain (MeV-Edm) and parainfluenza type 5 (PIV-5) as controls. PIV-5 does not activate the IFN pathway (Childs *et al.*, 2012), whilst SeV (Cantell strain) and MeV-Edm are known to be strong inducers (Shingai *et al.*, 2007; Strahle *et al.*, 2006; Takaki *et al.*, 2011). At 24 h after inoculation at an m.o.i. 3, the supernatants were tested for IFN content and compared with mock-inoculated cell supernatants. The supernatant of P2_low_-inoculated cells displayed similar low amounts of IFN to the supernatants of cells inoculated with PIV-5. In contrast, P5_high_-inoculated cells produced nearly as much IFN as SeV- or MeV-Edm-inoculated cells (Fig. 1). The lack of IFN induction by P2_low_ was not due to a lower infection efficiency, as FACS analysis 24 h after inoculation revealed infection of more than 90 % of the cells for all viruses. HMPV NL/1/00 thus only activated the IFN pathway when virus stocks were prepared at a high m.o.i. The ability of SeV, vesicular stomatitis virus (VSV), MeV-Edm and PIV-5 to strongly activate the IFN pathway has been associated with the presence of DIs in these virus stocks (Baum *et al.*, 2010; Killip *et al.*, 2013; Marcus & Sekellick, 1977; Sekellick & Marcus, 1982). In addition, studies with VSV revealed that the IFN-inducing capacity of DIs was not affected by UV treatment (Marcus & Gaccione, 1989). UV treatment of P5_high_, P2_low_ and SeV virus stocks resulted in reduced infection of A549 cells with P5_high_ and SeV but did not affect IFN induction by these viruses. This suggested that the IFN induction by P5_high_ may be attributed to the presence of DIs (Fig. S1, available in the online Supplementary Material).

### Deep sequencing demonstrates the presence of DIs

**Fig. 2.**
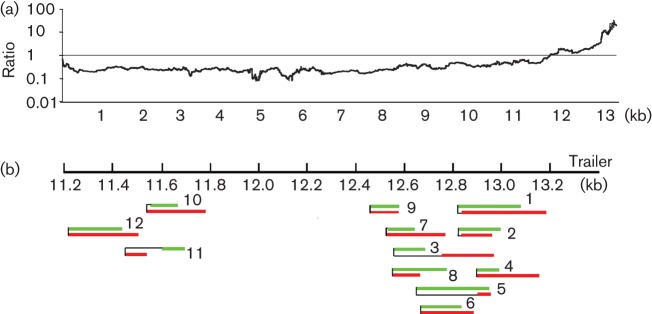
Deep sequencing of P2_low_ and P5_high_ demonstrating the presence of DIs in P5_high_. (a) Ratio between the coverage of depth per position between P2_low_ and P5_high_. The *x*-axis corresponds to the nt 13 365 position in the HMPV genome. (b) Sequences obtained for 12 proposed DIs were mapped against the HMPV genome. Green bars indicate the genomic strand, the red bars indicate the antigenomic strand and black lines indicate that the two bars were connected at that point.

To investigate the possible presence of DIs, RNA from virus stocks of P2_low_ and P5_high_ was subjected to arbitrarily primed 454 sequencing. Mapping of the sequence reads against the full-length HMPV genome resulted in 28 121 and 8200 sequence reads for P2_low_ and P5_high_, respectively. The genomes of P2_low_ and P5_high_ were covered with a mean of 588 and 168 reads per nucleotide, respectively. No coverage was obtained for the last 50 and 4 nt for P2_low_ and P5_high_, respectively. In addition, the ends of the genomes were covered with fewer than 10 reads from nt 13 164 for P2_low_ and from nt 13 325 for P5_high_. Fig. 2(a), depicting the ratio between the depths of sequence obtained for P2_low_ and for P5_high_ per nucleotide position, demonstrates that, at the end of the genome, P5_high_ displayed a greater depth of sequence than P2_low_. From nucleotide 11 726 onwards, P5_high_ displayed up to 31 times more depth of sequence than P2_low_. In fact, for P5_high_ 16.3 % of the 8200 reads mapped to the region beyond position 11 kb of the genome, compared with only 2.2 % of 28 121 reads obtained for P2_low_. Examination of the P5_high_ reads at this part of the genome revealed the presence of reads that only aligned for approximately 50 % with the reference sequence. Subsequent blast searches conducted with all sequence reads mapping for at least 20 % to the reference sequence revealed the presence of reads that contained two copies of (almost) the same genome region: one part of the read mapped to the genomic negative-sense sequence and the other part mapped to the antigenomic positive-sense sequence of the viral genome, with the two parts being complementary. In total, 47 dsRNA structures were identified in P5_high_ and none in P2_low_. All 47 reads aligned to the region upstream of position 11 200 of the viral genome, the same region where P5_high_ displayed deeper coverage than P2_low_. Alignment of these reads revealed 12 unique structures that differed in sequence and position, as shown in Fig. 2(b). As assignment of the orientation of the two strands could not be carried out based on the deep-sequencing results, the assignment was based on the results obtained in the blast search. The complementarity of the two strands indicated that P5_high_ contained DIs with a ‘snapback’ structure. The 12 DIs had variable lengths of the single-stranded parts at the cross-over point (i.e. the position where the polymerase switches from the template to the daughter strand and where the positive- and negative-sense strands of the DI are connected). No specific sequence motif was detected at the position of cross-over points that may have served as a signal for the polymerase to switch strands. Table S1 depicts the exact positions of all DIs compared with the viral genome.

### Detection and quantification of DIs by Northern blot assays

**Fig. 3.**
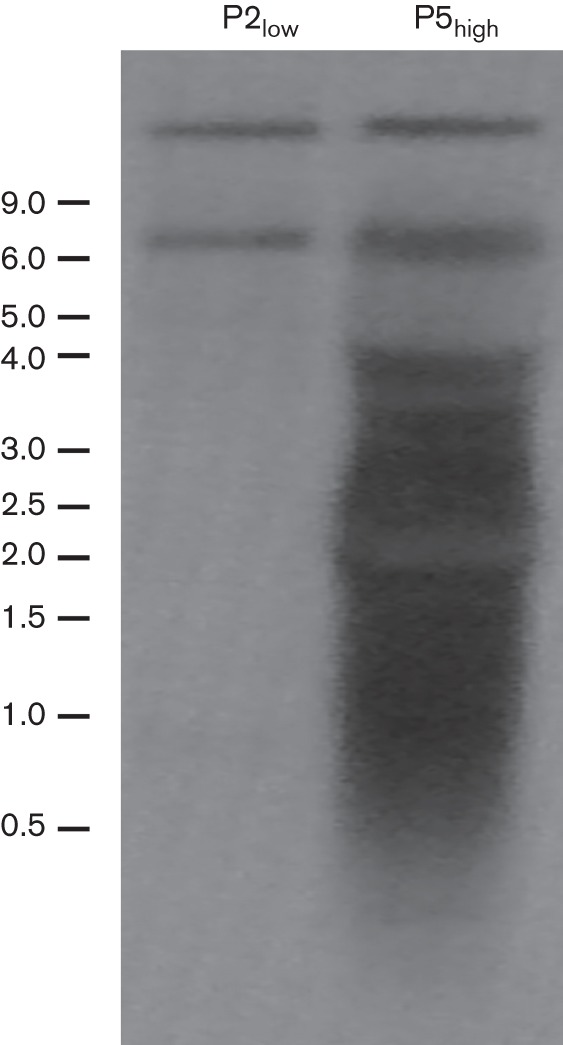
Northern blot analysis with a trailer probe showing the presence of small viral RNA. Upon RNA electrophoresis and hybridization with a probe complementary to the 5′ end of the genome (trailer), the blot was exposed to a phosphorimager screen and analysed with a Storm 850 PhosphorImager and ImageQuant 1.2 software. Size markers (kb) are indicated on the left.

To replicate, DIs need to contain polymerase initiation sites at the 5′ end (genomic/negative-sense strand) and the reverse complement of this trailer at the 3′ end (antigenomic/positive-sense strand). The deep-sequencing and bioinformatics approach only identified a read as a DI if the read overlapped with a cross-over point. All DI reads were too short to reach from the cross-over point to the trailer sequences or the reverse complement thereof. To test whether the double-stranded structures detected with deep sequencing were indeed DIs with a ‘snapback’ structure, we performed electrophoresis of viral RNA and Northern blotting using a probe complementary to the trailer. The Northern blot assay demonstrated the presence of similar amounts of full-length viral genome in P5_high_ and P2_low_ (Fig. 3, top band) with an additional band of approximately 7 kb detected in both virus stocks. The identity of the 7 kb fragment is unknown. In addition to these large RNA molecules, smaller RNAs ranging in size from 0.5 to 4 kb were detected but only in P5_high_. As the RNA was analysed on denaturing gels, fragments of this size may represent DIs, double-stranded in nature, of 0.25–2 kb. Quantification using a ChemiDoc Imaging system indicated that 80 % of the total RNA, in the size range 0.5–4 kb for P5_high_, was probably of DI origin.

In theory, the detection of dsRNA structures could have been a result of the process of deep sequencing, as this method involves ligation of linkers to PCR products. Reverse transcription (RT)-PCR assays directly on the virus stocks conducted with primers designed based on the consensus sequence obtained for DI#1 (Fig. S2) confirmed the presence of DIs in the original virus stocks. Besides DI#1, these RT-PCR assays identified additional DIs that were not identified during analysis of the 454 sequencing reads (Fig. S3).

### HMPV DI RNAs are hypermutated

**Fig. 4.**
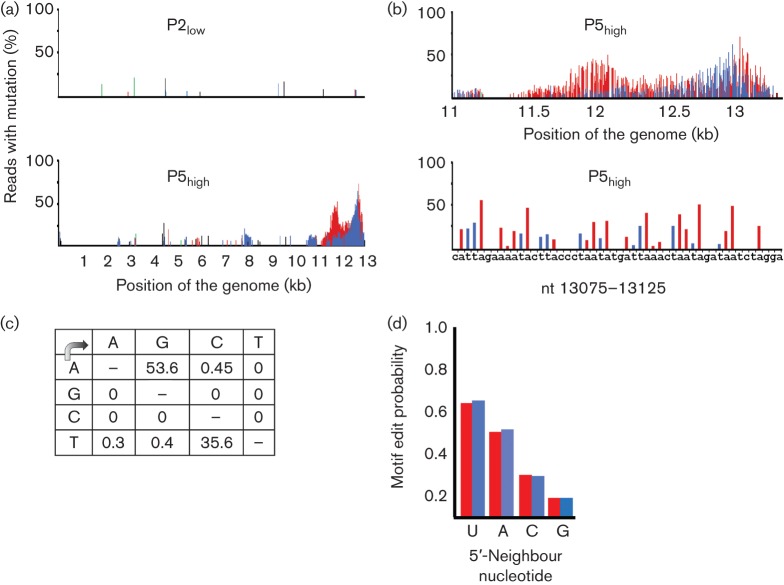
Mutations detected in the HMPV genome upon deep sequencing. (a) Percentage of reads with a particular mutation per nucleotide for the complete genome of P2_low_ and P5_high_. The x-axis corresponds to the 13 365 position in the HMPV genome, and the y-axis displays the percentage of reads that had a particular mutation, with a cut off set at 2% of the reads. (b) Detailed representation of the percentage of reads with a particular mutation per nucleotide for the regions of nt 11 000–13 365 (top) and nt 13 075–13 125 (bottom) for P5_high_. Green bars, mutation to A; black bars, mutation to T; blue bars, mutation to C; red bars, mutation to G. (c) Mutation matrices (%) for deep-sequencing reads obtained for the region downstream of nt 11 000 of the genome. Mutation was calculated when at least 5 % of reads displayed a mutation for that nucleotide. (d) Analysis of edited reads for the edit probability of an A or T, given a particular 5′-neighbour nucleotide. The highest probability for A→G or T→C editing was detected where the 5′-neighbour nucleotide was a U. Red bars indicate the A→G edit probability for the 5′ neighbour, and blue bars the T→C edit probability for the 3′ neighbour after complementing the neighbour with its base pair.

Analysis of the sequence variation of the deep-sequencing reads revealed extensive A→G and T→C mutations at the end of the genome of P5_high_ compared with the reference sequence, and this was not observed in P2_low_ (Fig. 4a). The location of hypermutations matched with the region where P5_high_ displayed more sequence depth than P2_low_ and where DIs were detected. In the region downstream of nt 11 000, 53.6 % of the A residues in the reference sequence displayed mutation to G and 35.7 % of the T residues displayed mutation to C (in at least 2 % of the reads mapping to that position) (Fig. 4b, c). In the region beyond position 12 000, 75 % of the reads displayed hypermutation and the mutation percentages in this region were even higher: 87.3 % A→G and 82.8 % T→C mutation. Individual reads varied in the position and number of mutations, with a range of 5–77 % of the A residues mutated to G and 5.7–57.1 % of the T residues mutated to C. We hypothesized that this kind of hypermutation was probably caused by the host RNA-editing enzyme ADAR. ADAR binds to dsRNA and deaminates A to I, which is recognized as a G during virus replication (Bass, 2002). Three human ADAR genes have been identified, and two of these have editing activity, ADAR1 and ADAR2.

### Role of ADAR in editing of DIs

We analysed whether the editing patterns in HMPV DIs were consistent with those described for ADAR editing by Eggington *et al.* (2011) who, refining earlier work by Lehmann & Bass (2000), determined that ADARs preferentially target As with a certain 5′ and a variable 3′ neighbour. They found that ADAR editing of A→I was more likely when the 5′ neighbour was U, followed by A, C and G (U>A>C>G). The 3′ preference was less specific, with a different preference for each of the four ADARs they considered and equal preference for some nucleotides. We analysed all reads (*n* = 954) mapping downstream of nt 11 000 for the editing probability depending on the 3′- and 5′-neighbour nucleotide. The mean edit probability for each of the 16 combinations of 5′ and 3′ neighbours of an edited A or T was calculated. No pattern was identified for the 3′-neighbouring nucleotide. In contrast, the 5′-neighbour preference was in accordance with the probabilities as described for ADAR (Eggington *et al.*, 2011), and was the same for A→G and T→C mutation (Fig. 4d).

In human cells, two isoforms of the ADAR1 protein are generated through the use of alternative promoters and alternative splicing. The long form (ADAR1-p150) is IFN inducible and localizes mostly in the cytoplasm, whereas a short form (ADAR1-p110) is expressed constitutively and localizes, like ADAR2, predominantly in the nucleus (George *et al.*, 2011). Vero cells are derived from African green monkeys, which might have different ADAR genes compared with human cells. RT-PCR assays performed with primer sets designed based on the human ADAR1 sequences in combination with the available African green monkey ADAR1 sequences detected mRNA of both ADARs in Vero-118 cells (Fig. S4a). Subsequent sequencing of the amplified fragments revealed that the Vero-ADAR1-p150 and Vero-ADAR-p110 mRNA displayed 95 % nucleotide sequence identity with the human ADAR1-p150 and ADAR1-p110 mRNAs and 99 % with those of macaques (Fig. S5). In addition, Western blotting using an antibody against human ADAR1 revealed expression of ADAR1-p110 and ADAR1-p150 in Vero-118 cells, and upon IFN treatment a slight increase in expression of ADAR1-p150 was observed (Fig. S4b).

Given the cytoplasmic replication of HMPV, the editing motifs and the expression of ADAR mRNA, we suggest that ADAR1 is the most likely enzyme responsible for editing of HMPV DIs.

## Discussion

As HMPV causes an acute respiratory infection in mammals it must – like other respiratory viruses – employ a mechanism to counteract the IFN response of the innate immune system. Several studies have reported interactions between HMPV and the innate immune system, although with conflicting conclusions. We did not observe induction of IFN production for HMPV NL/1/00 and NL/1/99 upon passage at low m.o.i. Only after a few passages at high m.o.i. was HMPV-induced IFN production observed. We showed that this induction was caused primarily by the presence of DIs with a ‘snapback’ structure in the virus stocks, providing a plausible explanation for the discrepancies in the data reported so far. The current study was conducted in Vero-118 cells. HMPV virus stocks have been generated in LLC-MK2 cells by other groups (Ren *et al.*, 2014; Velayutham *et al.*, 2013). However, in our hands, these cells do not produce sufficiently high viral titres to allow passage at high m.o.i. Future research needs to elucidate whether HMPV does generate DIs in LLC-MK2 cells or any other cells.

In theory, the detection of dsRNA structures could have been a result of the process of deep sequencing, as this method involves ligation of linkers to PCR products. However, no double-stranded structures were observed in P2_low_, which was analysed using the same protocol. In addition, both RT-PCR and Northern blots were performed using RNA isolated directly from the virus stock. Of note, the deep-sequencing analyses, Northern blots and RT-PCR assays performed using several independent P5_high_ stocks consistently revealed the presence of DIs and similar patterns of hypermutation. Our results indicated that different P5_high_ stocks contained various DIs.

Northern blot analyses revealed that the DIs ranged in size from 0.5 to 2 kb and represented more than 80 % of the RNA. Based on the Northern blot assays, the virus stock contained a larger number of DIs than detected with deep sequencing. As smaller RNAs transfer more efficiently compared with larger RNAs in Northern blotting, 80 % might be an overestimation. However, the deep sequencing may be less efficient in obtaining reads mapping to the 5′ end of the genome, which might result in an underestimation of the number of DI reads. In addition, many more reads in the deep-sequencing analysis might have been of DI origin but not identified as such as they lacked the cross-over point.

Analysis of mutations in the deep-sequencing reads obtained for P5_high_ and P2_low_ revealed the presence of A→G and T→C mutation only in P5_high_ and only in the region where DIs were identified. In addition, all reads that were identified as DI reads and the DI-PCR products displayed hypermutation. Although we cannot exclude hypermutation of the viral genome, the location of the observed hypermutation (in the polymerase gene) would, in that case, probably result in decreased virus replication. We observed similar replication of P2_low_ and P5_high_, arguing against hypermutation of the viral genome itself (Fig. S6).

So far, two mammalian RNA-editing enzymes are known: apolipoprotein B mRNA editing enzyme, catalytic polypeptide-like (APOBEC) and ADAR (Hamilton *et al.*, 2010). APOBEC deaminates C to U on ssDNA or RNA. APOBEC-induced specific C→U editing has been described for retroviruses and DNA viruses but not for RNA viruses (Bransteitter *et al.*, 2009). Commercial gene expression assays did not detect expression of APOBEC mRNA in our Vero-118 cells (data not shown), whilst we did detect ADAR mRNAs. ADAR binds to dsRNA and deaminates A to I, which, during replication, is recognized as a G (Bass, 2002). Three ADAR genes have been identified, of which two have editing activity, ADAR1 and ADAR2. The IFN-inducible ADAR1-p150 resides in the cytoplasm, whilst the constitutively expressed ADAR1-p110 and ADAR2 reside in the nucleus (George *et al.*, 2011). For viruses that replicate in the cytoplasm of the infected host cell, such as HMPV, ADAR1-p150 is the most likely protein responsible for A→I editing (Samuel, 2011). We did not observe a clear upregulation of ADAR1-p150 mRNA in IFN-treated Vero-118 cells, and only a low level of upregulation of protein in IFN-treated A549 and Vero cells. In accordance, in studies detecting expression of endogenous ADAR1-p150, only low expression levels were observed (Cavarec *et al.*, 2013). Vero-118 cells are IFN deficient (Desmyter *et al.*, 1968), which would argue against a role for ADAR1-p150. However, despite the absence of IFN genes in Vero cells, it is well known that expression of IFN-stimulated genes (e.g. ISG56) can be upregulated in an IFN-independent manner (Gélinas *et al.*, 2011). We did observe upregulation of ISG56 mRNA upon SeV or P5_high_ inoculation of Vero-118 cells (Fig. S7). Tripathi *et al.* (2014) found indications for IFN-independent induction of ADAR expression through the concerted action of IRF3 and IRF7, although the induction levels were low. However, the promoter that initiates the ADAR1-p150 transcript has a basal constitutive activity, which may be sufficient for editing activity (Patterson & Samuel, 1995).

ADAR-induced hypermutation of MeV has been studied in detail, but it is unclear why this hypermutation was not observed upon inoculation of Vero cells with MeV (Suspène *et al.*, 2011; Wong *et al.*, 1989). Although we have strong indications for the role of ADAR in HMPV-infected Vero cells, studies in ADAR-deficient cells and RNA-binding studies should elucidate the actual role of ADAR in editing HMPV (DI) RNA. The restricted host range of HMPV does not allow studies in ADAR-deficient MEFs or HeLa cells, and the low expression of ADAR will be a challenge for RNA-binding studies.

DIs have been demonstrated to play a role in the pathogenesis of some virus infections, such as in acute dengue virus and influenza virus infections (de Chassey *et al.*, 2013; Li *et al.*, 2011; Saira *et al.*, 2013). Given the relative ease with which HMPV accumulates DIs and the role demonstrated for DIs, the role that DIs may play in the pathogenesis of HMPV is clearly of interest.

In addition, DIs present in virus stocks have been shown to enable persistent infections both *in vivo* and *in vitro* (Cave *et al.*, 1985; Roux *et al.*, 1991; Weiss *et al.*, 1983). This is thought to be a result of activation of the innate immune system by DIs (Sekellick & Marcus, 1978). For HMPV, prolonged shedding of virus after experimental infection of mice has been reported (Alvarez *et al.*, 2004; Hamelin *et al.*, 2006; Liu *et al.*, 2009). It would be interesting to investigate whether the virus stocks used in these persistently infected animals contained DIs, as described for RSV (Estripeaut *et al.*, 2008; Mejías *et al.*, 2008).

Extensive A→I editing has been described in RNAs from MeV isolated from patients with persistent central nervous system infection (Cattaneo *et al.*, 1988; Wong *et al.*, 1989). Subsequent studies demonstrated that ADAR1 acts as a proviral host factor in the context of MeV infection (Toth *et al.*, 2009). In contrast, ADAR has an antiviral function in other virus infections (Samuel, 2011; Taylor *et al.*, 2005). Future research needs to elucidate the roll of ADAR in HMPV pathogenesis.

In conclusion, upon high m.o.i. passaging, HMPV accumulated DIs rapidly, and these DIs were responsible for a robust induction of IFN production. Our results should redirect research aiming to elucidate how HMPV subverts the innate immune system. Based on the high rate and the patterns of hypermutation, the preference of the 5′-neighbouring nucleotide and the expression of ADAR in Vero-118 cells, we suggest that HMPV DIs are edited by ADAR. Based on the fact that HMPV (DIs) replicates in the cytoplasm of infected cells, we speculate that HMPV (DI) genomes are hypermutated by ADAR1-p150.

## Methods

### 

#### Cells and viruses.

293-T cells and Vero-118 cells were grown as described previously (Herfst *et al.*, 2004). A549 cells were grown in Ham’s F-12 medium (Invitrogen) supplemented with 10 % FCS, High Clone (HC-FCS; Greiner Bio-One), with 100 IU penicillin ml^−1^, 100 µg streptomycin ml^−1^ and 2 mM glutamine (PSG). The generation of recombinant HMPV NL/1/00 has been described previously (Herfst *et al.*, 2004). After virus rescue, supernatants were used to inoculate Vero-118 cells in infection medium: Iscove’s modified Dulbecco’s medium (IMDM; Life Technologies) supplemented with PSG and 3.75 µg trypsin (BioWhittaker) ml^−1^. Upon reaching 80 % cytopathic effect, cells and virus-containing supernatants were harvested. One stock was generated with a maximum of two passages in Vero-118 cells at a low m.o.i. of 0.01, and a second stock was generated by five passages in Vero-118 cells at a high m.o.i. of 3. High-titre virus stocks of MeV-Edm (de Vries *et al.*, 2010) and PIV-5 (strain W3) were generated with an m.o.i of 0.03 in Vero-118 cells in IMDM supplemented with 3 % HC-FCS and PSG. After 4 days, cells and virus-containing supernatants were harvested. For MeV-Edm, PIV-5 and HMPV, harvested cells and virus cultures were centrifuged 5 min at 467 ***g*** and the cell-free supernatants were subsequently purified on sucrose gradients (60–30 %) and aliquots were stored at –80 °C. SeV strain Cantell was grown in 10-day-old embryonated chicken eggs at 37 °C for 48 h. The titre of the virus stocks was determined by end-point titration in Vero-118 cells and expressed as TCID_50_ ml^−1^. Inoculation of A549 cells was carried out in Ham’s F-12 supplemented with 2 % HC-FCS and PSG.

#### Bioassays for IFN.

The IFN content in supernatants was measured using the ISRE-firefly luciferase reporter construct as described previously (Patel *et al.*, 2013).

#### cDNA synthesis and PCR.

RNA isolation and cDNA synthesis were performed as described previously (van den Hoogen *et al.*, 2001). Amplification of DI genomes was performed with primers BF568, 5′-CTCTGCATTCCCTAGATTATC-3′ (based on wt sequence); BF593 , 5′-CCGCCGCCACAGAAGAATGGC-3′; and BF595 , 3′-GTTGACCATTTGGACTCTATGGCC-5′ (based on sequences obtained for DI#1) (Fig. 4a). Primers for ADAR-1 p150 were: 5′-GCGCAATGAATCCGCGGG-3′ (forward primer, based on human ADAR-1 p150; GenBank accession no. NM001111.4) and 5′-CCCCTGCCTTTCCATGTCAATTAGC-3′ (reverse primer, based on AGM ADAR-1 p150; GenBank accession no. NM001111.4). Primers for ADAR-1 p110 were: 5′-GAGAAGGCTACGTGGTGG-3′ (forward primer, based on human ADAR1 p110, George & Samuel, 1999) and 5′-AAAAAACTCAAGAGGATCTTCCAAGGC-3′ (reverse primer, end of exon 2; GenBank accession no. EF190455). Thermocycling was performed with the following conditions: 94 °C for 1 min, 40 °C for 2 min and 72 °C for 3 min (40 cycles). Gene expression assays were purchased from Applied Biosystems: Hs01020780-m1 for ADAR-1 p150, Hs01017596-m1 for ADAR-1 p110 and Hs00210562-m1 for ADAR-2. Real-time PCR assays were performed with a 7000 Sequence Detection System (Applied Biosystems), using Taqman Universal Mastermix according to the manufacturer’s instructions.

Arbitrarily primed PCR and virus genome sequencing were performed as described previously (van Boheemen *et al.*, 2012). GS Junior sequence reads were trimmed by 25 nt at both the 5′ and 3′ ends of the reads to remove primer sequences and aligned to the HMPV genome (GenBank accession no. AF371337) using CLC Genomics software v.4.6.1 (CLC bio). Using this software, single-nucleotide polymorphism analysis was conducted with a minimum of 2 % variation frequency and a minimal coverage of five reads per position.

#### Northern blot analysis.

RNA was isolated from purified virus stock (10^7^ TCID_50_ per sample) using TRIzol (Invitrogen) according to the manufacturer’s instructions. After electrophoresis of the RNA on a 1 % agarose gel, RNA was transferred to a positively charged nylon membrane (BrightStar-Plus; Ambion) and incubated with the probe. The probe, complementary to the 5′ end of genome, was generated with a Strip-EZ RNA system (Ambion) using a PCR fragment based on the full-length cDNA construct of NL/1/00 (Herfst *et al.*, 2004) with PCR primers 5′-TAATACGACTCACTATAGGGGAAAATGATAAAATG-3′ (forward primer, T7 promoter underlined) and 5′-GGTTTTTTTGCCGT-3′ (reverse complement to end of trailer). The blot was analysed with a NorthernMax-Gly system (Ambion), exposed to a phosphorimager screen and analysed with a Storm 850 PhosphorImager and ChemiDoc MP Imaging System and Image Lab v.4.0.1 software (Molecular Dynamics).

## References

[r1] Alvarez R. Harrod K. S. Shieh W. J. Zaki S. Tripp R. A. **(**2004**).** Human metapneumovirus persists in BALB/c mice despite the presence of neutralizing antibodies. J Virol 78, 14003–14011 10.1128/JVI.78.24.14003-14011.2004 15564507 PMC533920

[r2] Baños-Lara M. R. Ghosh A. Guerrero-Plata A. **(**2013**).** Critical role of MDA5 in the interferon response induced by human metapneumovirus infection in dendritic cells and in vivo. J Virol 87, 1242–1251 10.1128/JVI.01213-12 23152520 PMC3554051

[r3] Bao X. Liu T. Spetch L. Kolli D. Garofalo R. P. Casola A. **(**2007**).** Airway epithelial cell response to human metapneumovirus infection. Virology 368, 91–101 10.1016/j.virol.2007.06.023 17655903 PMC2266690

[r4] Bao X. Liu T. Shan Y. Li K. Garofalo R. P. Casola A. **(**2008**).** Human metapneumovirus glycoprotein G inhibits innate immune responses. PLoS Pathog 4, e1000077 10.1371/journal.ppat.1000077 18516301 PMC2386556

[r5] Bass B. L. **(**2002**).** RNA editing by adenosine deaminases that act on RNA. Annu Rev Biochem 71, 817–846 10.1146/annurev.biochem.71.110601.135501 12045112 PMC1823043

[r6] Baum A. Sachidanandam R. García-Sastre A. **(**2010**).** Preference of RIG-I for short viral RNA molecules in infected cells revealed by next-generation sequencing. Proc Natl Acad Sci U S A 107, 16303–16308 10.1073/pnas.1005077107 20805493 PMC2941304

[r7] Bransteitter R. Prochnow C. Chen X. S. **(**2009**).** The current structural and functional understanding of APOBEC deaminases. Cell Mol Life Sci 66, 3137–3147 10.1007/s00018-009-0070-y 19547914 PMC11115857

[r8] Cattaneo R. Schmid A. Eschle D. Baczko K. ter Meulen V. Billeter M. A. **(**1988**).** Biased hypermutation and other genetic changes in defective measles viruses in human brain infections. Cell 55, 255–265 10.1016/0092-8674(88)90048-7 3167982 PMC7126660

[r9] Cavarec L. Vincent L. Le Borgne C. Plusquellec C. Ollivier N. Normandie-Levi P. Allemand F. Salvetat N. Mathieu-Dupas E. **& other authors (**2013**).** In vitro screening for drug-induced depression and/or suicidal adverse effects: a new toxicogenomic assay based on CE-SSCP analysis of *HTR2C* mRNA editing in SH-SY5Y cells. Neurotox Res 23, 49–62 10.1007/s12640-012-9324-9 22528247

[r10] Cave D. R. Hendrickson F. M. Huang A. S. **(**1985**).** Defective interfering virus particles modulate virulence. J Virol 55, 366–373 2991562 10.1128/jvi.55.2.366-373.1985 PMC254942

[r11] Childs K. Randall R. Goodbourn S. **(**2012**).** Paramyxovirus V proteins interact with the RNA helicase LGP2 to inhibit RIG-I-dependent interferon induction. J Virol 86, 3411–3421 10.1128/JVI.06405-11 22301134 PMC3302505

[r12] de Chassey B. Aublin-Gex A. Ruggieri A. Meyniel-Schicklin L. Pradezynski F. Davoust N. Chantier T. Tafforeau L. Mangeot P. E. **& other authors (**2013**).** The interactomes of influenza virus NS1 and NS2 proteins identify new host factors and provide insights for ADAR1 playing a supportive role in virus replication. PLoS Pathog 9, e1003440 10.1371/journal.ppat.1003440 23853584 PMC3701712

[r13] de Vries R. D. Lemon K. Ludlow M. McQuaid S. Yüksel S. van Amerongen G. Rennick L. J. Rima B. K. Osterhaus A. D. **& other authors (**2010**).** In vivo tropism of attenuated and pathogenic measles virus expressing green fluorescent protein in macaques. J Virol 84, 4714–4724 10.1128/JVI.02633-09 20181691 PMC2863733

[r14] Desmyter J. Melnick J. L. Rawls W. E. **(**1968**).** Defectiveness of interferon production and of rubella virus interference in a line of African green monkey kidney cells (Vero). J Virol 2, 955–961 4302013 10.1128/jvi.2.10.955-961.1968 PMC375423

[r15] Eggington J. M. Greene T. Bass B. L. **(**2011**).** Predicting sites of ADAR editing in double-stranded RNA. Nat Commun 2, 319 10.1038/ncomms1324 21587236 PMC3113232

[r16] Estripeaut D. Torres J. P. Somers C. S. Tagliabue C. Khokhar S. Bhoj V. G. Grube S. M. Wozniakowski A. Gomez A. M. **& other authors (**2008**).** Respiratory syncytial virus persistence in the lungs correlates with airway hyperreactivity in the mouse model. J Infect Dis 198, 1435–1443 10.1086/592714 18828742 PMC3689551

[r17] Gélinas J. F. Clerzius G. Shaw E. Gatignol A. **(**2011**).** Enhancement of replication of RNA viruses by ADAR1 via RNA editing and inhibition of RNA-activated protein kinase. J Virol 85, 8460–8466 10.1128/JVI.00240-11 21490091 PMC3165853

[r18] George C. X. Samuel C. E. **(**1999**).** Human RNA-specific adenosine deaminase ADAR1 transcripts possess alternative exon 1 structures that initiate from different promoters, one constitutively active and the other interferon inducible. Proc Natl Acad Sci U S A 96, 4621–4626 10.1073/pnas.96.8.4621 10200312 PMC16382

[r19] George C. X. Gan Z. Liu Y. Samuel C. E. **(**2011**).** Adenosine deaminases acting on RNA, RNA editing, and interferon action. J Interferon Cytokine Res 31, 99–117 10.1089/jir.2010.0097 21182352 PMC3034097

[r20] Goutagny N. Jiang Z. Tian J. Parroche P. Schickli J. Monks B. G. Ulbrandt N. Ji H. Kiener P. A. **& other authors (**2010**).** Cell type-specific recognition of human metapneumoviruses (HMPVs) by retinoic acid-inducible gene I (RIG-I) and TLR7 and viral interference of RIG-I ligand recognition by HMPV-B1 phosphoprotein. J Immunol 184, 1168–1179 10.4049/jimmunol.0902750 20042593 PMC2834787

[r21] Hamelin M. E. Prince G. A. Gomez A. M. Kinkead R. Boivin G. **(**2006**).** Human metapneumovirus infection induces long-term pulmonary inflammation associated with airway obstruction and hyperresponsiveness in mice. J Infect Dis 193, 1634–1642 10.1086/504262 16703506

[r22] Hamilton C. E. Papavasiliou F. N. Rosenberg B. R. **(**2010**).** Diverse functions for DNA and RNA editing in the immune system. RNA Biol 7, 220–228 10.4161/rna.7.2.11344 20220309

[r23] Herfst S. de Graaf M. Schickli J. H. Tang R. S. Kaur J. Yang C. F. Spaete R. R. Haller A. A. van den Hoogen B. G. **& other authors (**2004**).** Recovery of human metapneumovirus genetic lineages A and B from cloned cDNA. J Virol 78, 8264–8270 10.1128/JVI.78.15.8264-8270.2004 15254198 PMC446134

[r24] Huang A. S. **(**1973**).** Defective interfering viruses. Annu Rev Microbiol 27, 101–118 10.1146/annurev.mi.27.100173.000533 4356530

[r25] Killip M. J. Young D. F. Gatherer D. Ross C. S. Short J. A. Davison A. J. Goodbourn S. Randall R. E. **(**2013**).** Deep sequencing analysis of defective genomes of parainfluenza virus 5 and their role in interferon induction. J Virol 87, 4798–4807 10.1128/JVI.03383-12 23449801 PMC3624313

[r26] Kolakofsky D. **(**1976**).** Isolation and characterization of Sendai virus DI-RNAs. Cell 8, 547–555 10.1016/0092-8674(76)90223-3 182384

[r27] Lazzarini R. A. Keene J. D. Schubert M. **(**1981**).** The origins of defective interfering particles of the negative-strand RNA viruses. Cell 26, 145–154 10.1016/0092-8674(81)90298-1 7037195

[r28] Lehmann K. A. Bass B. L. **(**2000**).** Double-stranded RNA adenosine deaminases ADAR1 and ADAR2 have overlapping specificities. Biochemistry 39, 12875–12884 10.1021/bi001383g 11041852

[r29] Li D. Lott W. B. Lowry K. Jones A. Thu H. M. Aaskov J. **(**2011**).** Defective interfering viral particles in acute dengue infections. PLoS ONE 6, e19447 10.1371/journal.pone.0019447 21559384 PMC3084866

[r30] Liao S. Bao X. Liu T. Lai S. Li K. Garofalo R. P. Casola A. **(**2008**).** Role of retinoic acid inducible gene-I in human metapneumovirus-induced cellular signalling. J Gen Virol 89, 1978–1986 10.1099/vir.0.2008/000778-0 18632970 PMC2865242

[r31] Liu Y. Haas D. L. Poore S. Isakovic S. Gahan M. Mahalingam S. Fu Z. F. Tripp R. A. **(**2009**).** Human metapneumovirus establishes persistent infection in the lungs of mice and is reactivated by glucocorticoid treatment. J Virol 83, 6837–6848 10.1128/JVI.00379-09 19357175 PMC2698565

[r32] Marcus P. I. Gaccione C. **(**1989**).** Interferon induction by viruses. XIX. Vesicular stomatitis virus—New Jersey: high multiplicity passages generate interferon-inducing, defective-interfering particles. Virology 171, 630–633 10.1016/0042-6822(89)90637-5 2474895

[r33] Marcus P. I. Sekellick M. J. **(**1977**).** Defective interfering particles with covalently linked [±]RNA induce interferon. Nature 266, 815–819 10.1038/266815a0 194158

[r34] Mejías A. Chávez-Bueno S. Gómez A. M. Somers C. Estripeaut D. Torres J. P. Jafri H. S. Ramilo O. **(**2008**).** Respiratory syncytial virus persistence: evidence in the mouse model. Pediatr Infect Dis J 27 (Suppl.), S60–S62 10.1097/INF.0b013e3181684d52 18820580

[r35] Patel J. R. Jain A. Chou Y. Y. Baum A. Ha T. García-Sastre A. **(**2013**).** ATPase-driven oligomerization of RIG-I on RNA allows optimal activation of type-I interferon. EMBO Rep 14, 780–787 10.1038/embor.2013.102 23846310 PMC3790048

[r36] Patterson J. B. Samuel C. E. **(**1995**).** Expression and regulation by interferon of a double-stranded-RNA-specific adenosine deaminase from human cells: evidence for two forms of the deaminase. Mol Cell Biol 15, 5376–5388 7565688 10.1128/mcb.15.10.5376 PMC230787

[r37] Preston F. M. Straub C. P. Ramirez R. Mahalingam S. Spann K. M. **(**2012**).** siRNA against the G gene of human metapneumovirus. Virol J 9, 105 10.1186/1743-422X-9-105 22676157 PMC3393630

[r38] Randall R. E. Goodbourn S. **(**2008**).** Interferons and viruses: an interplay between induction, signalling, antiviral responses and virus countermeasures. J Gen Virol 89, 1–47 10.1099/vir.0.83391-0 18089727

[r39] Ren J. Liu G. Go J. Kolli D. Zhang G. Bao X. **(**2014**).** Human metapneumovirus M2-2 protein inhibits innate immune response in monocyte-derived dendritic cells. PLoS ONE 9, e91865 10.1371/journal.pone.0091865 24618691 PMC3950292

[r61] Rima B. K. Davidson W. B. Martin S. J. **(**1977**).** The role of defective interfering particles in persistent infection of Vero cells by measles virus. J Gen Virol 35, 89–97 404394 10.1099/0022-1317-35-1-89

[r40] Roux L. Simon A. E. Holland J. J. **(**1991**).** Effects of defective interfering viruses on virus replication and pathogenesis in vitro and in vivo. Adv Virus Res 40, 181–211 10.1016/S0065-3527(08)60279-1 1957718 PMC7131706

[r41] Saira K. Lin X. DePasse J. V. Halpin R. Twaddle A. Stockwell T. Angus B. Cozzi-Lepri A. Delfino M. **& other authors (**2013**).** Sequence analysis of in vivo defective interfering-like RNA of influenza A H1N1 pandemic virus. J Virol 87, 8064–8074 10.1128/JVI.00240-13 23678180 PMC3700204

[r42] Samuel C. E. **(**2011**).** Adenosine deaminases acting on RNA (ADARs) are both antiviral and proviral. Virology 411, 180–193 10.1016/j.virol.2010.12.004 21211811 PMC3057271

[r43] Sekellick M. J. Marcus P. I. **(**1978**).** Persistent infection I. Interferon-inducing defective-interfering particles as mediators of cell sparing: possible role in persistent infection by vesicular stomatitis virus. Virology 85, 175–186 10.1016/0042-6822(78)90422-1 206002

[r44] Sekellick M. J. Marcus P. I. **(**1982**).** Interferon induction by viruses. VIII. Vesicular stomatitis virus: [±]DI-011 particles induce interferon in the absence of standard virions. Virology 117, 280–285 10.1016/0042-6822(82)90530-X 6175087

[r45] Shingai M. Ebihara T. Begum N. A. Kato A. Honma T. Matsumoto K. Saito H. Ogura H. Matsumoto M. Seya T. **(**2007**).** Differential type I IFN-inducing abilities of wild-type versus vaccine strains of measles virus. J Immunol 179, 6123–6133 10.4049/jimmunol.179.9.6123 17947687

[r46] Spann K. M. Tran K. C. Collins P. L. **(**2005**).** Effects of nonstructural proteins NS1 and NS2 of human respiratory syncytial virus on interferon regulatory factor 3, NF-κB, and proinflammatory cytokines. J Virol 79, 5353–5362 10.1128/JVI.79.9.5353-5362.2005 15827150 PMC1082743

[r47] Strahle L. Garcin D. Kolakofsky D. **(**2006**).** Sendai virus defective-interfering genomes and the activation of interferon-β. Virology 351, 101–111 10.1016/j.virol.2006.03.022 16631220

[r48] Suspène R. Petit V. Puyraimond-Zemmour D. Aynaud M. M. Henry M. Guétard D. Rusniok C. Wain-Hobson S. Vartanian J. P. **(**2011**).** Double-stranded RNA adenosine deaminase ADAR-1-induced hypermutated genomes among inactivated seasonal influenza and live attenuated measles virus vaccines. J Virol 85, 2458–2462 10.1128/JVI.02138-10 21159878 PMC3067779

[r49] Takaki H. Watanabe Y. Shingai M. Oshiumi H. Matsumoto M. Seya T. **(**2011**).** Strain-to-strain difference of V protein of measles virus affects MDA5-mediated IFN-β-inducing potential. Mol Immunol 48, 497–504 10.1016/j.molimm.2010.10.006 21071089

[r50] Taylor D. R. Puig M. Darnell M. E. Mihalik K. Feinstone S. M. **(**2005**).** New antiviral pathway that mediates hepatitis C virus replicon interferon sensitivity through ADAR1. J Virol 79, 6291–6298 10.1128/JVI.79.10.6291-6298.2005 15858013 PMC1091666

[r51] Toth A. M. Li Z. Cattaneo R. Samuel C. E. **(**2009**).** RNA-specific adenosine deaminase ADAR1 suppresses measles virus-induced apoptosis and activation of protein kinase PKR. J Biol Chem 284, 29350–29356 10.1074/jbc.M109.045146 19710021 PMC2785566

[r52] Tripathi S. Nsosie E. O. Brownstein J. S. Garcia-Sastre A. **(**2014**).** Characterization of interferon independent induction of host antiviral genes. In *Innate Immunity to Viral Infections*, Poster J6-3044. Keystone Symposia, 19–24 January, Keystone, Colorado USA.

[r53] van Boheemen S. de Graaf M. Lauber C. Bestebroer T. M. Raj V. S. Zaki A. M. Osterhaus A. D. Haagmans B. L. Gorbalenya A. E. **& other authors (**2012**).** Genomic characterization of a newly discovered coronavirus associated with acute respiratory distress syndrome in humans. MBio 3, e00473-12 10.1128/mBio.00473-12 23170002 PMC3509437

[r54] van den Hoogen B. G. de Jong J. C. Groen J. Kuiken T. de Groot R. Fouchier R. A. Osterhaus A. D. **(**2001**).** A newly discovered human pneumovirus isolated from young children with respiratory tract disease. Nat Med 7, 719–724 10.1038/89098 11385510 PMC7095854

[r55] van den Hoogen B. G. Bestebroer T. M. Osterhaus A. D. Fouchier R. A. **(**2002**).** Analysis of the genomic sequence of a human metapneumovirus. Virology 295, 119–132 10.1006/viro.2001.1355 12033771

[r56] van den Hoogen B. G. Herfst S. Sprong L. Cane P. A. Forleo-Neto E. de Swart R. L. Osterhaus A. D. Fouchier R. A. **(**2004**).** Antigenic and genetic variability of human metapneumoviruses. Emerg Infect Dis 10, 658–666 10.3201/eid1004.030393 15200856 PMC3323073

[r57] Velayutham T. S. Kolli D. Ivanciuc T. Garofalo R. P. Casola A. **(**2013**).** Critical role of TLR4 in human metapneumovirus mediated innate immune responses and disease pathogenesis. PLoS ONE 8, e78849 10.1371/journal.pone.0078849 24205331 PMC3812158

[r58] Weiss B. Levis R. Schlesinger S. **(**1983**).** Evolution of virus and defective-interfering RNAs in BHK cells persistently infected with Sindbis virus. J Virol 48, 676–684 6632084 10.1128/jvi.48.3.676-684.1983 PMC255400

[r59] Williams J. V. Harris P. A. Tollefson S. J. Halburnt-Rush L. L. Pingsterhaus J. M. Edwards K. M. Wright P. F. Crowe J. E. Jr **(**2004**).** Human metapneumovirus and lower respiratory tract disease in otherwise healthy infants and children. N Engl J Med 350, 443–450 10.1056/NEJMoa025472 14749452 PMC1831873

[r60] Wong T. C. Ayata M. Hirano A. Yoshikawa Y. Tsuruoka H. Yamanouchi K. **(**1989**).** Generalized and localized biased hypermutation affecting the matrix gene of a measles virus strain that causes subacute sclerosing panencephalitis. J Virol 63, 5464–5468 2585612 10.1128/jvi.63.12.5464-5468.1989 PMC251217

